# Molecular Characterization of Lineage IV Peste des Petits Ruminants Viruses in Sudan Isolated from Outbreaks Between 2015 and 2018 Suggests the Existence of the North–East Africa Episystem

**DOI:** 10.3390/v18070765

**Published:** 2026-07-12

**Authors:** Rayan M. Asil, Arnaud Bataille, Martin Ludlow, Albert D. M. E. Osterhaus, Abdelgadir Ballal, Salma O. Abdelgadir, Muzdalifa A. Hassan, Yahia H. Ali, Saafass Alsarraj, Felix Njeumi, Satya Parida, Nussieba A. Osman

**Affiliations:** 1 Department of Pathology, Parasitology and Microbiology, College of Veterinary Medicine, Sudan University of Science and Technology, Khartoum-North P.O. Box 204, Sudan; rayanasil89@gmail.com; 2 Central Veterinary Research Laboratory (CVRL), Virology Department, Khartoum P.O. Box 8067, Sudan; vet.salmaahmed@gmail.com (S.O.A.); muzdalifa73@yahoo.com (M.A.H.); y_beik@hotmail.com (Y.H.A.); 3 CIRAD, UMR ASTRE, 34398 Montpellier, France; arnaud.bataille@cirad.fr; 4 ASTRE, University of Montpellier, CIRAD, INRAE, 34398 Montpellier, France; 5 Research Center for Emerging Infections and Zoonoses, University of Veterinary Medicine Hannover, 30559 Hannover, Germany; martin.ludlow@tiho-hannover.de (M.L.); albert.osterhaus@tiho-hannover.de (A.D.M.E.O.); 6 Central Veterinary Research Laboratory (CVRL), Viral Vaccine Production Department, Khartoum P.O. Box 8067, Sudan; 7 Food and Agriculture Organization of the United Nations (FAO), Viale delle Terme di Caracalla, 00153 Rome, Italy; felix.njeumi@fao.org (F.N.); satya.parida@fao.org (S.P.); 8 Royal Veterinary College, University of London, Hawkshead Lane, North Mymms, Hatfield, London AL9 7TA, UK

**Keywords:** peste des petits ruminants (PPR), small ruminants, Sudan, outbreaks, nucleoprotein gene, *Morbillivirus*, phylogenetics, episystem

## Abstract

Multiple outbreaks with high mortality rates and a pattern of disease occurrence similar to peste des petits ruminants (PPR) were observed in sheep and goats across many states of Sudan between 2015 and 2018. Therefore, this study aimed to investigate and identify the cause of these disease outbreaks. A total of 276 blood and lung tissue samples were collected from infected sheep (n = 223) and goats (n = 53). Sample analysis in an IC-ELISA revealed the presence of the PPRV antigen in 56.9% of sheep and 62.3% of goat samples. Twenty-seven [sheep (18) and goats (9)] PPRV-positive samples from IC-ELISA were subsequently confirmed positive using the PPRV N-gene-based RT-PCR. Two PPR viruses were isolated from infected small ruminant lung tissues in Vero cells. The partial N-gene sequences for five PPRV strains originating from sheep and goats were determined. Phylogenetic tree grouped PPRV strains identified in this study in lineage IV in sub-lineage NEA (North–East Africa), with the highest sequence identity observed with strains circulating in North African countries. This study and earlier published phylogenetic analysis and patterns of animal movements suggest that the transboundary transmission of PPRV lineage IV strains between East and North African countries has been happening since 2008 and established a new North–East Africa episystem that needs to be disrupted for the successful global eradication of PPR.

## 1. Introduction

Peste des petits ruminants (PPR) is an acute, highly contagious transboundary viral disease affecting domesticated small ruminants and wild Artiodactyl species. Sheep and goats, which serve as primary hosts, are naturally susceptible to peste des petits ruminants virus (PPRV) infection and play a crucial epidemiological role in maintaining virus endemicity in regions of Africa and Asia [1,2,3]. Disease progression in infected animals is variable, ranging from peracute or acute to chronic [3,4]. Clinical PPR is characterized by the rapid onset of depressive symptoms, fever, disordered eating, serous and mucopurulent discharges from the eyes and nose, erosion of the mouth, necrotic stomatitis with gingivitis, conjunctivitis, foul-smelling diarrhea, cough, bronchopneumonia, and death [3,4]. PPRV infections are typically acute and life-threatening, resulting in elevated morbidity and mortality rates [5,6,7]. The global community recognizes PPR as a major barrier for small ruminant production and food security in low- and middle-income countries [8,9,10].

The World Organization for Animal Health (WOAH, formerly the OIE) has assigned PPR as a notifiable disease caused by PPRV, which was recently classified as the only member of the *Morbillivirus caprinae* species within the *Morbillivirus* genus of the *Paramyxoviridae* family [11,12]. The viral genome is 15,948 nucleotides in length and is made up of a non-segmented, single-stranded, negative-sense RNA molecule that adheres to the “rule of six” (multiple of six) [13]. The PPRV genome comprises six non-overlapping transcription units that encode six structural proteins [the nucleoprotein (N), the phosphoprotein (P), the matrix protein (M), the fusion protein (F), the haemagglutinin (H) and the polymerase (L)], and two nonstructural proteins [C and V proteins] [13,14]. Although PPRV has a single serotype, molecular surveillance studies based on partial sequences of the F gene [15,16] or the N gene [17,18] have shown that there are four genetic lineages (I, II, III, and IV) with differing geographical distributions.

PPR was first detected in Sudan in 1971, when outbreaks of a Rinderpest-like disease were observed in small ruminants in three locations of Gedarif (eastern Sudan) [19]. Outbreaks of the disease were also documented in Sinnar (south-eastern Sudan) from 1971 until 1972, as well as in Mieliq (central Sudan) in 1972 [19]. At that time, the disease was incorrectly diagnosed as Rinderpest (ovine RP). Nonetheless, the disease was later shown to be PPR, following the identification of two strains of PPRV (SUD 72/1, Sinnar strain; SUD 72/2, Mieliq strain), which had been isolated from samples obtained from goats during outbreaks in 1971 and 1972 [20]. New disease outbreaks were observed in Elhilalia, Gezira State, from 1989 to 1990, and a PPR virus strain VHL was isolated [21]. Subsequently, cases of PPRV were regularly reported in sheep and goats across Sudan [22,23,24,25,26,27,28,29,30], with additional sporadic PPR cases noted in camels [31,32] and gazelles [33]. Serological surveys regularly reported PPRV seroconversion, indicating previous virus infection in various animal species, in almost every Sudanese state [24,25,26,29,34,35,36,37,38,39,40,41,42,43,44,45]. PPR is now one of the highest-risk diseases affecting small ruminants in Sudan, with endemic transmission of PPRV occurring in different regions of Africa. North Africa was free of PPR up to 2008 when Morocco reported its first outbreak [46,47]. Soon after that, Algeria and Tunisia reported outbreaks in their countries in the year 2011 [47,48,49,50]. In response to the ongoing outbreaks in Sudan and to support the episystem-based PPR global eradication program (PPR GEP) initiative, the current status of this disease and characterization of circulating viral strains need to be updated for each endemic country. Consequently, in this study, we have identified, isolated, and characterized PPRV strains that have circulated in small ruminant herds in Sudan to establish the relationship of circulating viruses in the country as well as in the neighboring countries in East and North Africa.

## 2. Materials and Methods

### 2.1. PPR Outbreaks and Study Area

**Figure 1:**
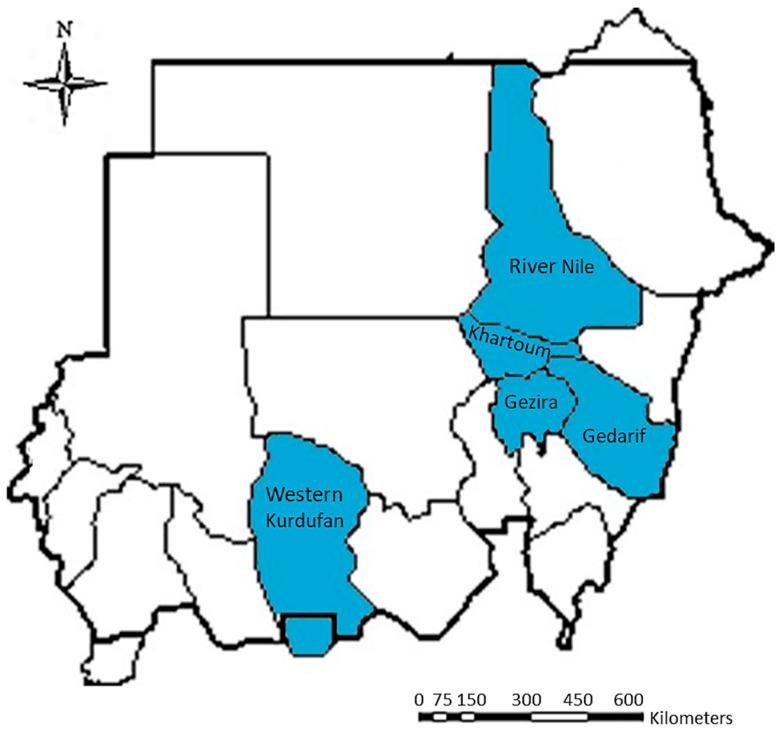
Geographical locations of five different Sudanese States from where clinical samples were collected from sheep and goats.

From 2015 to 2018, suspected PPR outbreaks occurred among sheep and goats in five Sudanese states; Khartoum State [2015–2018] and Gezira State [2016–2017] in central Sudan; River Nile State [2016–2017] in northern Sudan; Gedarif State [2015–2017] in eastern Sudan; and Western Kurdufan State [2017] in western Sudan (Figure 1). A total of 33 outbreaks have been documented in these Sudanese states (Table 1). Farmers who noticed the clinical symptoms that accompanied fatalities were the first to report incidents related to PPR to the local veterinary authority. The local veterinary authority then reported the incident to the national veterinary authority and the General Directorate of Epizootic Disease Control, Khartoum.

### 2.2. Sample Collection and Preparation

In Sudan, 276 samples were collected from sheep (223 = 216 whole blood and 7 lung tissues) and goats (53 = 50 whole blood and 3 lung tissues) with suspected PPR clinical signs (Table 1). These samples were collected by veterinarians and virologists from the General Directorate of Epizootics Disease Control and veterinary research laboratories in Sudanese States and delivered to the CVRL in Khartoum for laboratory diagnosis. Samples were collected from Khartoum, Gezira, River Nile, Gedarif, and Western Kurdufan States (Figure 1). In the center of Sudan, samples were collected from Khartoum State (142 samples, 136 whole blood, and 6 lung tissues from 109 sheep and 33 goats) and Gezira State (82 whole blood samples from 66 sheep and 16 goats). In northern Sudan, only samples from River Nile State were collected (32 whole blood samples from 28 sheep and 4 goats). The eastern Sudan samples were obtained from Gedarif State (4 lungs from 4 sheep), while the western Sudan samples originated from Western Kurdufan State (16 whole blood samples from 16 sheep) (Table 1; Figure 1). Whole blood samples had been stored at 4 °C, and the buffy coat was separated and stored at −20 °C before use. A 20% lung tissue homogenate was prepared in phosphate-buffered saline (PBS) with antibiotics (Penicillin–Streptomycin) (Sigma-Aldrich, Burlington, MA, USA) and antimycotics (Mycostatin) (Sigma-Aldrich, Burlington, MA, USA) and stored at −20 °C until use.

### 2.3. Immunocapture ELISA (IC-ELISA) Assay

To establish a diagnosis, the buffy coat and lung homogenate of sheep and goats were examined for the presence of PPRV antigen using the ID Screen^®^ PPR Antigen Capture IC-ELISA kit (IDVet, Grabels, France) according to the manufacturer’s instructions.

### 2.4. Cell Culture, Virus Isolation and Identification

Isolation of PPRV was performed on Vero cells maintained in Glasgow minimum essential medium (GMEM) (Sigma-Aldrich, Burlington, MA, USA) supplemented with 5% fetal bovine serum (Sigma-Aldrich, Burlington, MA, USA). Virus isolation was attempted using the Buffy coat, swab supernatants, and 20% tissue homogenate as virus inoculum on Vero cell monolayers. Following infection, infected Vero cells were investigated daily under an inverted microscope for the appearance of PPRV-specific cytopathic effect (CPE). Furthermore, the cell culture medium was changed every 3 days; occasionally, to prevent overgrowth, the infected cells were subjected to subculturing and blind passaging by freeze-thawing the cell monolayer. Upon observation of CPE involving 90% of the cell monolayer at 27 days post-infection, the infected cells and viral supernatants were collected and preserved at −70 °C. The presence of PPR viruses in the infected cell culture supernatants was confirmed using the described IC-ELISA. Further, PPRV strain identification was performed by an RT-PCR assay, using PPRV N-gene-specific primers, and N-gene Sanger sequencing.

### 2.5. RNA Extraction and Reverse Transcription–Polymerase Chain Reaction (RT-PCR)

The reference PPRV Nigeria 75/1 vaccine strain of PPRV lineage II [51,52] served as a positive viral control in RT-PCR. The vaccine was obtained from CVRL’s Viral Vaccine Production Department in Khartoum, Sudan.

Total viral RNA was purified directly from whole blood buffy coat samples using the RNeasy^®^ Mini Kit (Qiagen, Hilden, Germany), and RNA was extracted from lung tissues and cell culture supernatants of the PPRV Nigeria 75/1 vaccine strain using the QIAamp Viral RNA^®^ Mini Kit (Qiagen, Hilden, Germany) according to the manufacturer’s instructions. For molecular characterization of PPRV, the partial PPRV N-gene sequence was amplified using a Qiagen One-Step RT-PCR Kit (Qiagen, Hilden, Germany) and N-gene-specific primers set [NP3 forward primer (5′-TCT CGG AAA TCG CCT CAC AGA CTG-3′) and NP4 reverse primer (5′-CCT CCT CCT GGT CCT CCA GAA TCT-3′)] obtained from Eurofins Genomics, Germany [17]. The RT-PCR cycling conditions included cDNA synthesis in 1 cycle of reverse transcription at 50 °C for 30 min, RT inactivation and initial polymerase activation at 95 °C for 15 min, followed by 40 cycles of cDNA amplification corresponding to denaturation at 95 °C for 10 s, annealing at 55 °C for 30 s, extension at 72 °C for 30 s, and finally 1 cycle for PCR reaction termination by heating at 72 °C for 5 min. Electrophoresis of 5 μL PCR product on a 1.5% agarose gel revealed an expected amplicon size of 351 bp.

### 2.6. Sanger Sequencing and Data Analysis

The partial sequence of the PPRV N-gene [the 3′ end: 1232–1583 nt] for samples found positive in both the IC-ELISA and RT-PCR was determined by Sanger sequencing. The generated PCR amplicons were subjected to sequencing in an automated DNA sequencer using a DNA sequencing kit (BGI Genomics, Shenzhen, China). The resulting sequences of the PPRV partial N-gene from the sheep and goat samples were assembled using CodonCode Aligner (https://www.codoncode.org/), and any overlapping and primer sequences were removed. These sequences were compared to N-gene sequences of PPRV strains available in the NCBI GenBank database using Nucleotide BLAST (http://blast.ncbi.nlm.nih.gov/; accessed on 25 June 2026) to identify the percentages of nucleotide sequence identity with other PPRV strains.

### 2.7. Phylogenetic Analysis for PPRV Lineage Identification

The partial PPRV N-gene sequences (351 nt) obtained from sheep and goat samples were aligned with the relevant N-gene sequences of other PPRV strains available in the GenBank database using the ClustalW multiple alignment program (http://www.ebi.ac.uk/Tools/msa/clustalw2/; accessed on 1 December 2025). To identify PPRV lineages, a phylogenetic analysis of the aligned PPRV N-gene partial sequences was performed using the MEGA12 (Molecular Evolutionary Genetics Analysis) software version 12 (www.megasoftware.net/) [53]. A phylogenetic tree was generated using the neighbor-joining method, and the distances were calculated using the Maximum Composite Likelihood approach [54]. Bootstrap confidence intervals were determined for 1000 replicates with clusters supported by bootstrap values greater than 50 indicated at the nodes of the resulting phylogenetic tree.

## 3. Results

### 3.1. Clinical Manifestations of PPR in Sheep and Goats

**Figure 2:**
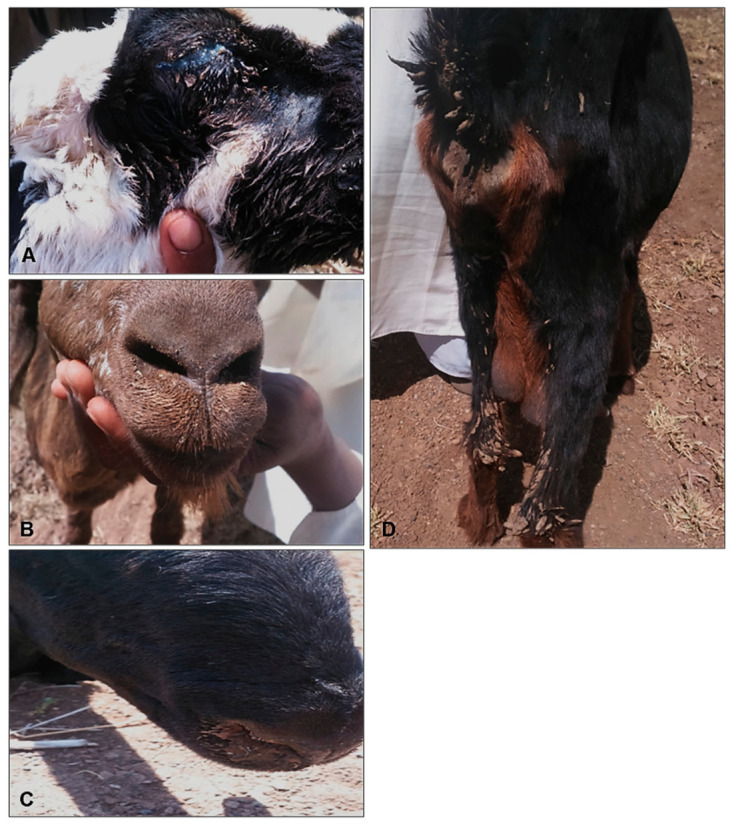
Clinical signs of PPR in sheep and goats in Gezira State during 2016. (**A**) Ocular discharges; (**B**,**C**) nasal discharges; (**D**) diarrhoea in a goat.

PPRV infection was suspected in these outbreaks of disease in sheep and goats, with clinical signs including pyrexia, anorexia, profuse serous nasal and ocular discharges, while some animals displayed mucopurulent discharges, dry muzzle, cough, signs of pneumonia, diarrhoea, and death (Figure 2). Comparatively, goats showed more severe symptoms than sheep. Suspected PPR cases were connected to increased morbidity and mortality rates, particularly among groups of young animals.

### 3.2. IC-ELISA for the Detection of PPRV Antigen in Samples Collected from Sheep and Goats

Of the 276 sheep and goat samples collected from PPR suspected cases analyzed by IC-ELISA, 160 (58%) were found to be PPRV-positive, demonstrating a high PPRV-antigen presence. Considering the animal species, samples from 127 (56.9%) out of 223 sheep and 33 (62.3%) of the 53 goats were PPRV-positive, indicating a higher antigenic presence in goats compared to sheep (Table 2).

Considering the type of samples examined by IC-ELISA, 154/266 (57.9%) whole blood samples from both small ruminant species were PPRV-positive. PPRV was identified in 123/216 (56.9%) of sheep whole blood samples and 31/50 (62%) of goat whole blood samples. Assessment of lung tissues from sheep and goats using IC-ELISA showed that 6/10 (60%) were PPRV-positive. PPRV was identified in 4/7 (57.1%) sheep lung tissues and 2/3 (66.7%) goat lung tissues.

The percentage of positivity of PPRV antigen in suspected samples collected from five states of Sudan was determined by IC-ELISA (Table 2). Within the states that were under investigation, Western Sudan [Western Kurdufan State] showed the highest percentage of positivity (81.2%) followed by Northern Sudan [River Nile State (65.6%)]. Middle Sudan [Gezira State (58.5%) and Khartoum State (53.5%)] showed a moderate positivity while Eastern Sudan [Gedarif State (50%)] had the lowest positivity. To investigate the percentage positivity among animal species in five Sudanese states studied, the highest positivity among sheep was found in Western Kurdufan State (81.2%) followed by River Nile State (64.3%), Gezira State (62.1%), Gedarif State (50%), and Khartoum State (48.6%) (Table 2). The highest percentage positivity among goats (75%) was observed in River Nile State, followed by Khartoum State (69.7%), while Gezira State had the lowest percentage positivity (43.8%) (Table 2).

### 3.3. PPRV Isolation and Identification

Virus isolation was attempted on multiple occasions, but PPRV was successfully isolated only twice in Vero cells inoculated with 10% tissue homogenate. Following subculturing and blind passaging, at 15 days post-infection (d.p.i.), infected Vero cells began to exhibit cytopathic effects (CPEs) indicative of PPRV, including cell rounding, followed by gradual monolayer detaching, reaching 80–90% at 25–27 days post-infection. Uninfected Vero cells were treated identically as a control. Two PPRV isolates, “PPRV/tc/Sudan/Khartoum/2015” and “PPRV/tc/Sudan/Gedarif/2015”, were recovered from infected goat and sheep lung tissues, respectively. IC-ELISA, RT-PCR assays and N-gene sequencing confirmed the identity of these PPR viral isolates.

### 3.4. Reverse Transcription–Polymerase Chain Reaction (RT-PCR) for Detection of PPRV RNA

Twenty-seven sheep and goat samples from PPR suspected outbreaks that were confirmed positive by IC-ELISA were selected and further characterized using a PPRV N-gene-based RT-PCR assay. All 27 (100%) samples investigated yielded the PPRV N-gene with bands of 351 bp. The RT-PCR-positive samples comprised 18/27 (66.7%) and 9/27 (33.3%) samples from sheep and goats, respectively (Table 2). The remaining samples were not tested due to a lack of materials for RNA extraction and RT-PCR.

### 3.5. Amplicon Sequencing

The identity of each of the PPRV strains was determined by Sanger sequencing of RT-PCR amplicons. Five samples, one from each state, were selected to determine the PPRV partial N-gene sequence. These samples originated from sheep and goats that were positive for both IC-ELISA and PPRV N-gene-based RT-PCR. The 351 nt partial PPRV N-gene sequences of the five Sudanese PPRV strains were compiled and deposited in the NCBI GenBank database under accession numbers MK371448.1 (PPRV/Sudan/Khartoum/2015); MK371449.1 (PPRV/Sudan/Gedarif/2015); MK371450.1 (PPRV/Sudan/Gezira/Kab-Elgidad/2016); MK371451.1 (PPRV/Sudan/River-Nile/Garie/2016); and MK371455.1 (PPRV/Sudan/Western-Kurdufan/Abuzabad/2017) (Table 3).

### 3.6. Sequence Analysis

Partial PPRV N-gene sequences from sheep and goat samples were analyzed and compared to the respective N-gene sequences of PPRV strains available in the NCBI GenBank database using BLASTn (http://blast.ncbi.nlm.nih.gov/; accessed on 25 June 2026) to determine the percentages of nucleotide sequence identities with other PPRV strains. The PPRV sequences obtained in this study were found to be closely related to those of PPRV lineage IV strains. The five sequences generated in this study can be divided into two groups. There were three sequences in Group 1 (PPRV/Sudan/Khartoum/2015, PPRV/Sudan/Gezira/Kab-Elgidad/2016, and PPRV/Sudan/Western-Kurdufan/Abuzabad/2017) which were 100% identical. Similarly, the two sequences in Group 2 (PPRV/Sudan/Gedarif/2015 and PPRV/Sudan/River-Nile/Garie/2016) were 100% identical. The percentage of identity between Groups 1 and 2 is 99.15%. There were three nucleotide differences between Groups 1 and 2 which are located at positions 143 (G replaced by A), 158 (T replaced by C), and 188 (A replaced by G).

All sheep and goat PPR viruses from five Sudanese States shared the highest nucleotide sequence identity of 100% (group 1) and 98.58% (group 2) when compared with strain Georgia/G1/2016 (KY646062.1) while 99.00–99.43% identity was observed when compared with other European strains [Georgia/Tbilisi/2016 (MF737202.1), Greece/Elassona/adis5/2024 (PV385049.1), Romania/Tulcea/adis1/7/2024 (PV385050.1), Romania/adis1/13/2024 (PV385051.1); Romania/Tulcea/ADIS1/4/2024 (PQ642763.1)], Egyptian PPRV strains [Ismailia/1/Egypt/2010 (JN202923.2), Ismailia/3/Egypt/2010 (JN202924.2), Egypt/Ismailia/2010 (JN202925.1 and JN202926.1), PPRV/Sharkia/1/2019, PPRV/Sharkia/2/2019, PPRV/Sharkia/3/2019 and PPRV/Sharkia/4/2019 (MZ605284.1, MZ605285.1, MZ605286.1, MZ605287.1)], and Ethiopia/2011 (MK991798.1). The percentages of identity for sequences of Groups 1 and 2 are presented in the Supplementary Materials (Table S1).

### 3.7. Phylogenetic Analysis

**Figure 3:**
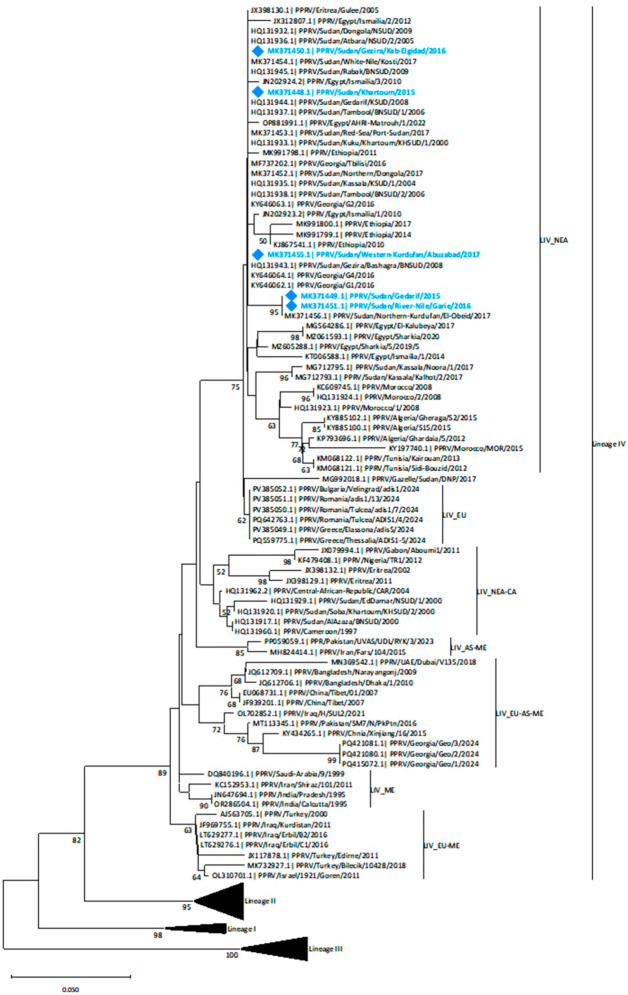
Phylogenetic tree using the neighbor-joining method for partial N-gene sequences (351 nt) of PPRV strains. Analysis was conducted in MEGA12 (Molecular Evolutionary Genetics Analysis) (www.megasoftware.net/) by analyzing 1000 bootstrap replicates, and clusters were supported by bootstrap values >50 (PPRV Sudanese Strains from sheep and goats represented by trquise blue font and diamonds). The neighbor-joining tree focuses on PPRV lineage IV which contains many sub-lineages where recent Sudanese PPR sequences are grouped within the North–East Africa (LIV_NEA) sub-lineage. Other lineage IV sub-lineages including Europe (LIV_EU), North–East Africa and Central Africa (LIV_NEA-CA), Asia (LIV_AS), Asia–Middle East (LIV_AS-ME), Europe–Asia–Middle East (LIV_EU-AS-ME), Middle East (LIV-ME) and Europe–Middle East (LIV-EU-ME).

A phylogenetic tree was constructed using partial N-gene sequences from the NCBI database originating from Sudan and neighboring countries in East and North Africa, revealing that the five PPRV sequences from Sudanese sheep and goats generated in this study belonged to the PPRV lineage IV and are closely related to PPRV sequences from North African countries and previously published viral sequences from Sudan (Figure 3). Notably, lineage IV contains several geographically separated sub-lineages [55,56], and the sequences obtained in this study were grouped within the North–East Africa (LIV_NEA) sub-lineage. Within this sub-lineage, the five sequences appear to be grouped into two different clades (Figure 3). Two sequences, PPRV/Sudan/Gedarif/2015 and PPRV/Sudan/River-Nile/Garie/2016 shared an identical sequence in the specific region of the N gene under investigation and were clustered together with a sequence obtained in Northern Kurdufan in 2017 with a bootstrap value of 95. The remaining three sequences, PPRV/Sudan/Khartoum/2015, PPRV/Sudan/Gezira/Kab-Elgidad/2016, and PPRV/Sudan/Western-Kurdufan/Abuzabad/2017, were clustered with sequences from Sudan, other North and East African countries, and European strains from Georgia (2016) with limited phylogenetic resolution (Figure 3).

## 4. Discussion

PPR has been periodically detected in Sudan since the early 1970s, with PPRV currently considered as endemically circulating across countries in the region. This endangers domestic small ruminants, which are considered the primary source of meat for human consumption besides cattle. In Sudan, small ruminants are raised in mixed herds as part of traditional breeding practices. According to the official reports from the Federal Ministry of Animal Resources and Fisheries, the estimated numbers of small ruminants in Sudan in the years 2015, 2016 and 2017 were 40,210, 40,612 and 40,752 sheep in addition to 31,230, 31,481 and 31,659 goats. The estimated numbers of sheep and goats were higher in Kurdufan followed by Gezira, and the lowest numbers were found in Khartoum.

Despite the use of the PPR live-attenuated vaccine [52] in some areas, especially after the initiation of the Sudan National PPR control program, multiple outbreaks of a small ruminant disease clinically identified as PPR have taken place in recent years across the country, suggesting that vaccination strategies implemented were ineffective. However, with the ongoing civil war in Sudan, it is impossible to vaccinate animals in unsafe places. Between 2015 and 2018, there were numerous suspected PPR outbreaks among sheep and goats in various Sudanese states. These outbreaks were marked primarily by the presence of typical PPR clinical symptoms in small ruminants, as well as elevated mortality and morbidity rates. According to data from the World Animal Health Information System [30], PPR outbreaks have occurred on a regular basis in the five states over the previous ten years. Therefore, a study was conducted to genetically characterize the virus strains at the origin of the disease outbreaks observed in domestic small ruminants in Sudan.

Using an IC-ELISA, in this study, an overall positive percentage of PPRV antigen of 58% was demonstrated among sheep and goats from PPR suspected outbreaks. In 2008, sheep in Sudan showed a lower percentage of PPRV antigen (42.6%) than in earlier studies [24]. Another study found a relatively low percentage of PPRV antigen in samples from sheep (15.4%) and goats (21.1%) from slaughterhouses and disease outbreaks [25]. From 2015 to 2017, researchers conducted a study on twelve suspected PPR outbreaks in sheep and goats across four localities in Kassala State (eastern Sudan). A Sandwich ELISA revealed a greater PPR antigen [28]. In this study, testing of whole blood samples from suspected sheep and goats for PPRV antigen using an IC-ELISA showed a 57.9% positive antigenic percentage. Additionally, PPRV was found in 62% and 56.9% of all blood samples from goats and sheep. Screening of lung tissues from sheep and goats found a 60% antigenic percentage, with PPRV discovered in 66.7% and 57.1% of goat and sheep lung tissues, respectively. It is evident that PPRV-positive antigen percentage is higher in lung tissue samples than in whole blood samples, indicating a higher viral detection rate in lung tissue samples than in other types of samples. In an earlier study, PPRV antigen was identified in 11%, 35%, and 30% of swab samples, tissue samples, and whole blood samples using an IC-ELISA assay [57], which is consistent with the results of the present study. Only limited comparisons can be done with results from multiple studies if they have not used the same sampling methodology.

In the five states where outbreaks of symptomatic PPR occurred, western Sudan experienced the highest positive PPRV-antigen percentage (81.2%), followed by northern Sudan (65.6%), middle Sudan (58.5–53.5%), and eastern Sudan (50%). This conclusion is consistent with those reported by [24], who reported the highest rate in western Sudan (62.5%) and the lowest percentage in middle Sudan (27.3%). Conversely, a recent study revealed an even higher antigenic percentage in eastern Sudan (73.3%) [28]. Regional differences in the percentage of PPRV-positive antigen were apparent with the highest percentage observed in west Sudan, with comparatively low percentages in north and middle Sudan and the lowest percentage in east Sudan. This variation in disease occurrence could be attributed to regional differences in vaccination coverage, symptomatic disease occurrence, animal movement, and herd immunity, but bias due to sampling design cannot be ruled out.

PPRV nucleic acid was identified by RT-PCR from 27 whole blood and lung samples from sheep and goats from suspected PPR outbreaks in five Sudanese states that tested positive by IC-ELISA. This finding is consistent with earlier studies that have shown the high sensitivity of an RT-PCR using NP3/NP4 primers to detect PPR nucleic acids [58,59]. Genetic characterization studies, based on the partial PPRV N-gene sequence, clustered older PPRV Sudanese isolates, from the early 1970s (PPRV SUD 71 Gedarif, PPRV SUD 72/1 Sinnar, and PPRV SUD 72/2 Mieliq strains), as well as a few new isolates after 2000, into the PPRV lineage III genotype along with East African and Arabian Peninsula isolates [32,60]. Interestingly, nearly all the new PPRV strains recovered from Sudan after the year 2000 were classified as PPRV lineage IV, suggesting gradual replacement of lineage III by IV in the country [27,28,32,33,56]. In this study, molecular typing of five PPR viruses from sheep and goat samples from five different Sudanese states confirmed the presence of PPRV lineage IV, which has been circulating in Sudan since 2000 [32]. PPRV N-gene sequences from Dorcas gazelle samples from Khartoum and Dinder National Park, Sudan, were also clustered into lineage IV with close similarity with strains from neighboring countries [33]. Similarly, six strains were identified from Kassala State in eastern Sudan as PPRV lineage IV using the partial N-gene sequence [28]. A recently published study [56] identified four strains from slaughtered small ruminants across four Sudanese states—Northern (northern), Red-Sea (eastern), White-Nile (central), and Northern Kurdufan (western)—which shared a high percentage identity with previous Sudanese viruses, as well as with strains from neighboring African countries (Eritrea, Ethiopia, Egypt) as stated in earlier studies [47,61]. The genetic similarity between Sudanese PPRV strains from sheep and goats and PPRV strains from North and some East African countries such as Egypt, Tunisia, Morocco, Algeria, and Ethiopia, forming a geographically well-defined, North–East Africa sub-lineage [47,55,56], supports the transboundary transmission of PPRV in the region, thus implying the need for an episystem regional control strategy of the disease. PPR emerged in East Africa, initially identified in Sudan in 1971–1972 [19], with subsequent reports from Egypt [62], Ethiopia [63], Eritrea [64], and Chad [65]. During the 2000s, the disease spread to North Africa, starting with Morocco [66], followed by Algeria [48], Tunisia [67], and Libya [68]. This pattern of disease report suggests a flow from Sudan to neighboring East African countries and then into North African countries. This data, generated from viral sequence analysis, is well supported by the animal movement study conducted by FAO and represented in a map in the study of Bazazi et al. [47], indicating that animals and PPRV are moving from East Africa to North Africa to establish a PPR episystem. Therefore, this study fully agrees with the earlier study [47] that for the effective eradication of PPR, a regional harmonized approach needs to be employed in this region. However, full genome sequence analyses on a larger dataset from Sudan and neighboring countries may be beneficial to further investigate the evolutionary history of PPRV in this region.

## 5. Conclusions

In summary, the detection of PPRV in several samples from suspected live or dead animals using IC-ELISA and RT-PCR indicates that PPRV lineage IV was the cause for the outbreaks and associated fatalities in small ruminants in Sudan during the 2015–2018 outbreaks. Phylogenetic analysis of partial N-gene sequences revealed that the PPRV strains identified in this study were grouped in different clades within sub-lineage NEA of lineage IV, with the highest sequence identity observed with strains originating from North and some of the East African countries. Considering the similarities of PPRV strains circulating in the East and North African region and the animal movement patterns in the region, a well-defined North–East Africa episystem-based eradication program should be prioritized by the members of countries and international organizations like AU-IBAR, WOAH and FAO.

## Figures and Tables

**Table 1:** Samples collected from sheep and goats from PPR suspected outbreaks in five states of Sudan.

Date of Collection	Place of Collection	No. of Outbreaks	Total No. of Samples	Type and Number of Samples					
				Sheep			Goats		
				Total No.	WB	L	Total No.	WB	L
2015–2018	Khartoum State	18	142	109	106	3	33	30	3
2016–2017	Gezira State	8	82	66	66	-	16	16	-
2016–2017	River Nile State	2	32	28	28	-	4	4	-
2015–2017	Gedarif State	4	4	4	-	4	-	-	-
2017	Western Kurdufan State	1	16	16	16	-	-	-	-
	Total	33	276	223	216	7	53	50	3

Notes: WB = whole blood, L = lung tissue.

**Table 2:** IC-ELISA and RT-PCR for detecting PPRV antigen/RNA in sheep and goat samples collected from PPR suspected outbreaks that occurred in five different states of Sudan.

Animal Species	Sheep and Goats			Sheep			Goats		
Methods	IC-ELISA		RT-PCR	IC-ELISA		RT-PCR	IC-ELISA		RT-PCR
Place of collection	Total No. tested (%)	No. +ve (%)	No. tested/+ve (%)	No. tested (%)	No. +ve (%)	No. tested/+ve (%)	No. tested (%)	No. +ve (%)	No. tested/+ve (%)
Khartoum State	142 (100%)	76 (53.5%)	10 (37.0%)	109 (100%)	53 (48.6%)	6 (60.0%)	33 (100%)	23 (69.7%)	4 (40.0%)
Gezira State	82 (100%)	48 (58.5%)	8 (29.6%)	66 (100%)	41 (62.1%)	5 (62.5%)	16 (100%)	7 (43.8%)	3 (37.5%)
River Nile State	32 (100%)	21 (65.6%)	3 (11.1%)	28 (100%)	18 (64.3%)	1 (33.3%)	4 (100%)	3 (75.0%)	2 (66.7%)
Gedarif State	4 (100%)	2 (50.0%)	2 (7.4%)	4 (100%)	2 (50.0%)	2 (100%)	-	-	-
Western Kurdufan State	16 (100%)	13 (81.2%)	4 (14.9%)	16 (100%)	13 (81.2%)	4 (100%)	-	-	-
Total	276 (100%)	160 (58%)	27 (100%)	223 (100%)	127 (56.9%)	18 (66.7%)	53 (100%)	33 (62.3%)	9 (33.3%)

**Table 3:** Peste des petits ruminants virus strains sequenced from sheep and goat samples from PPR outbreaks in Sudan.

Strain Name	Date of Collection	Place of Collection		Species	Type of Sample	Source	Group No.	Sequence Accession No.
		State	Locality					
PPRV/Sudan/Khartoum/2015	2015	Khartoum	Khartoum	Goat	Lung	Field	1	MK371448.1
PPRV/Sudan/Gedarif/2015	2015	Gedarif	Gedarif	Sheep	Lung	Field	2	MK371449.1
PPRV/Sudan/Gezira/Kab-Elgidad/2016	2016	Gezira	Kab Elgidad	Goat	Whole blood	Field	1	MK371450.1
PPRV/Sudan/River-Nile/Garie/2016	2016	River Nile	Garie	Sheep	Whole blood	Field	2	MK371451.1
PPRV/Sudan/Western-Kurdufan/Abuzabad/2017	2017	Western Kurdufan	Abuzabad	Sheep	Whole blood	Field	1	MK371455.1

Notes: Group 1 = 3 sequences are 100% identical; group 2 = 2 sequences are 100% identical. The percentage of identity between groups 1 and 2 is 99.15%.

## Data Availability

PPRV N-gene sequences of Sudanese strains from this study were deposited in the National Center for Biotechnology Information (NCBI) GenBank database (http://www.ncbi.nlm.nih.gov/; accessed on 31 December 2019) under accession numbers MK371448.1, MK371449.1, MK371450.1, MK371451.1 and MK371455.1.

## Supplementary Materials

The following supporting information can be downloaded at https://www.mdpi.com/article/10.3390/v18070765/s1, Table S1: Percentages of identity between Sudanese PPRV strains and other PPRV strains using the N-gene (351 nt) and BLAST nucleotides.

## Abbreviations

The following abbreviations are used in this manuscript:

AS	Asia
BLAST	Basic Local Alignment Search Tool
CA	Central Africa
CPE	Cytopathic effect
d.p.i.	Day post-infection
EU	Europe
EURL-PPR	Reference Laboratory of the European Union for Peste des Petits Ruminants
F gene	Fusion protein gene
GMEM	Glasgow minimum essential medium
IC-ELISA	Immunocapture ELISA
ICTV	International Committee on Taxonomy of Viruses
ME	Middle East
N gene	Nucleoprotein gene
NCBI	National Center for Biotechnology Information
NEA	North–East Africa
Nt	Nucleotide
OIE	Office International des Epizootics
PPR	Peste des petits ruminants
PPR GEP	PPR global eradication program
PPRV	Peste des petits ruminants virus
RP	Rinderpest
RT-PCR	Reverse transcription–polymerase chain reaction
SUD	Sudan
TC	Tissue culture
WAHIS	World Animal Health Information System
WOAH	World Organization for Animal Health
