# Supplementary material for: Molecular Characterization of Lineage IV Peste des Petits Ruminants Viruses in Sudan Isolated from Outbreaks Between 2015 and 2018 Suggests the Existence of the North–East Africa Episystem
Source: Viruses. 2026 Jul 12;18(7):765. doi: 10.3390/v18070765 (PMC13431533; doi:10.3390/v18070765)
Supplement: Supplementary file 1 [file viruses-18-00765-s001.zip › viruses-4325508-supplementary.pdf]

Supplementary materials

Table S1. Percentages of identity between Sudanese PPRV strains and other PPRV strains using the N-gene (351 nt) and BLAST nucleotides.

Table S1-1. Sudanese sequences group 1

Strain name	Percentage of identity	Length	Accession
PPRV/Sudan/Khartoum/2015	100	351	MK371448.1
PPRV/Georgia/G1/2016	100	320	KY646062.1
PPRV/Georgia/Tbilisi/2016	99.43	15948	MF737202.1
PPRV/Ismailia/1/Egypt/2010	99.43	351	JN202923.2
PPRV/Ismailia/3/Egypt/2010	99.43	354	JN202924.2
PPRV/Egypt/Ismailia/2010	99.41	337	JN202926.1
PPRV/Romania/Tulcea/ADIS1-4/2024	99.4	331	PQ642763.1
PPRV/Sharkia/2/2019	99.38	325	MZ605285.1
PPRV/Romania/adis1/13/2024	99.15	15948	PV385051.1
PPRV/Romania/Tulcea/adis1/7/2024	99.15	15948	PV385050.1
PPRV/Ethiopia/2011	99.15	15948	MK991798.1
PPRV/Greece/Elassona/adis5/2024	99.15	15948	PV385049.1
PPRV/Sudan/Gedarif/2015	99.15	351	MK371449.1
PPRV/Egypt/Ismailia/2010	99.14	353	JN202925.1
PPRV/Sharkia/4/2019	99.09	328	MZ605287.1
PPRV/Sharkia/3/2019	99.08	327	MZ605286.1
PPRV/Sharkia/1/2019	99.08	325	MZ605284.1
PPRV/Ethiopia/2010	98.86	15948	KJ867541.1
PPRV/AHRI-Matrouh/1	98.79	330	OP881991.1
PPRV/Sharkia/5/2019	98.78	327	MZ605288.1
PPRV/Ismailia/2/Egypt/2012	98.53	339	JX312807.1
PPRV/Ethiopia/Habru/2014	98.29	15948	ON110960.1
PPRV/Ethiopia/2014	98.29	15948	MK991799.1
PPRV/El-Kalubeya/2017	98.01	351	MG564286.1
PPRV/Sharkia/Egy/2020	97.99	348	MZ061593.1
PPRV/Ismailia/1/2014	97.72	1575	KT006588.1
PPRV/Gazelle/Sudan/DNP2017	97.72	351	MG992018.1
PPRV/Gazelle/Sudan/Khartoum/2016/1	97.72	351	MG992016.1
PPRV/Ethiopia/2017	97.71	15948	MK991800.1
PPRV/Reji/A2/2010	97.41	348	OL690338.1
PPRV/Algeria/S15/2015	97.15	15948	KY885100.1

Table S1-2. Sudanese sequences group 2

Strain name	Percentage of identity	Length	Accession
PPRV/Sudan/Gedarif/2015	100	351	MK371449.1
PPRV/Sudan/Khartoum/2015	99.15	351	MK371448.1

PPRV/Georgia/G1/2016	99.06	320	KY646062.1
PPRV/Georgia/Tbilisi/2016	98.58	15948	MF737202.1
PPRV/Ismailia/1/Egypt/2010	98.58	351	JN202923.2
PPRV/Ismailia/3/Egypt/2010	98.58	354	JN202924.2
PPRV/Egypt/Ismailia/2010	98.52	337	JN202926.1
PPRV/Romania/Tulcea/ADIS1-4/2024	98.49	331	PQ642763.1
PPRV/Sharkia/2/2019	98.46	325	MZ605285.1
PPRV/Ethiopia/2011	98.29	15948	MK991798.1
PPRV/Greece/Elassona/adis5/2024	98.29	15948	PV385049.1
PPRV/Romania/adis1/13/2024	98.29	15948	PV385051.1
PPRV/Romania/Tulcea/adis1/7/2024	98.29	15948	PV385050.1
PPRV/Egypt/Ismailia/2010	98.29	353	JN202925.1
PPRV/Sharkia/3/2019	98.17	327	MZ605286.1
PPRV/Sharkia/4/2019	98.17	328	MZ605287.1
PPRV/Sharkia/1/2019	98.15	325	MZ605284.1
PPRV/Ethiopia/2010	98.01	15948	KJ867541.1
PPRV/AHRI-Matrouh/1	97.88	330	OP881991.1
PPRV/Sharkia/5/2019	97.86	327	MZ605288.1
PPRV/Ismailia/2/Egypt/2012	97.64	339	JX312807.1
PPRV/Ethiopia/2014	97.44	15948	MK991799.1
PPRV/Gazelle/Sudan/Khartoum/2016/1	97.44	351	MG992016.1
PPRV/Ethiopia/Habru/2014	97.44	15948	ON110960.1
PPRV/El-Kalubeya/2017	97.15	351	MG564286.1
PPRV/Sharkia/Egy/2020	97.13	348	MZ061593.1
